# Oxymatrine ameliorates white matter injury by modulating gut microbiota after intracerebral hemorrhage in mice

**DOI:** 10.1111/cns.14066

**Published:** 2022-12-22

**Authors:** Jing Li, Jianhao Liang, Meiqin Zeng, Kaijian Sun, Yunhao Luo, Huaping Zheng, Feng Li, Wen Yuan, Hongwei Zhou, Junshan Liu, Haitao Sun

**Affiliations:** ^1^ Clinical Biobank Center, Microbiome Medicine Center, Department of Laboratory Medicine, Zhujiang Hospital Southern Medical University Guangzhou China; ^2^ Neurosurgery Center, The National Key Clinical Specialty, The Engineering Technology Research Center of Education Ministry of China on Diagnosis and Treatment of Cerebrovascular Disease, Guangdong Provincial Key Laboratory on Brain Function Repair and Regeneration, The Neurosurgery Institute of Guangdong Province, Zhujiang Hospital Southern Medical University Guangzhou China; ^3^ Laboratory Animal Center, Zhujiang Hospital Southern Medical University Guangzhou China; ^4^ Guangdong Provincial Key Laboratory of Chinese Medicine Pharmaceutics, School of Traditional Chinese Medicine Southern Medical University Guangzhou China; ^5^ Department of Pharmacy, Zhujiang Hospital Southern Medical University Guangzhou China; ^6^ Key Laboratory of Mental Health of the Ministry of Education, Guangdong‐Hong Kong‐Macao Greater Bay Area Centre for Brain Science and BrainInspired Intelligence Southern Medical University Guangzhou China

**Keywords:** fecal microbiota transplantation, gut microbiota, intracerebral hemorrhage, oxymatrine, white matter injury

## Abstract

**Introduction:**

White matter injury (WMI) significantly affects neurobehavioral recovery in intracerebral hemorrhage (ICH) patients. Gut dysbiosis plays an important role in the pathogenesis of neurological disorders. Oxymatrine (OMT) has therapeutic effects on inflammation‐mediated diseases. Whether OMT exerts therapeutic effects on WMI after ICH and the role of gut microbiota involved in this process is largely unknown.

**Methods:**

Neurological deficits, WMI, gut microbial composition, intestinal barrier function, and systemic inflammation were investigated after ICH. Fecal microbiota transplantation (FMT) was performed to elucidate the role of gut microbiota in the pathogenesis of ICH.

**Results:**

OMT promoted long‐term neurological function recovery and ameliorated WMI in the peri‐hematoma region and distal corticospinal tract (*CST*) region after ICH. ICH induced significant and persistent gut dysbiosis, which was obviously regulated by OMT. In addition, OMT alleviated intestinal barrier dysfunction and systemic inflammation. Correlation analysis revealed that gut microbiota alteration was significantly correlated with inflammation, intestinal barrier permeability, and neurological deficits after ICH. Moreover, OMT‐induced gut microbiota alteration could confer protection against neurological deficits and intestinal barrier disruption.

**Conclusions:**

Our study demonstrates that OMT ameliorates ICH‐induced WMI and neurological deficits by modulating gut microbiota.

## Graphical abstract

Schematic mechanism of the protection of OMT against gut dysbiosis and white matter injury (WMI) in ICH. ICH induced severe WMI and neurological deficits, accompanied by persistent gut dysbiosis. OMT promoted better long‐term neurological function recovery and ameliorated WMI after ICH by modulating gut microbial composition and alleviating intestinal barrier dysfunction.

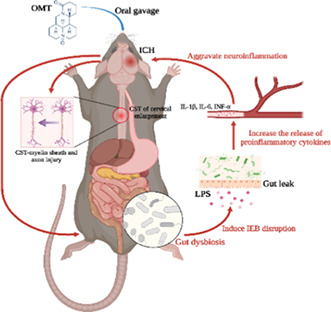

## 1 INTRODUCTION

Intracerebral hemorrhage (ICH) is the second most common subtype of stroke (10%–15%) with high morbidity and mortality, remaining a major cause of death and disability globally.^1^ A series of pathological changes, such as the mass effect of hematoma‐ and hemorrhage‐induced secondary injury, including neuronal death, demyelination, axonal degeneration, and glial scarring in the peri‐hematoma area, occur after ICH.^2^ White matter consists of axons, myelin sheaths, and supporting glial cells. Due to its crucial role in neural signal transmission, both subcortical white matter injury (WMI) and distal white matter fiber tracts disruption produce severe neurological deficits.^3^, ^4^ A clinical study reported that WMI was a common pathological event in patients with lobar ICH (present in 77% of patients),^5^ which is highly related to the motor impairment.^6^ Interestingly, WMI displays potential repairable property, which raises emerging attention from more researchers. However, more attention was paid to the progression and repairment of axonal degeneration and demyelination around primary brain lesions, and little was known about the pathological changes in whiter matter fiber tracts of the spinal cord, especially the corticospinal tract (*CST*), as well as its role in neurobehavioral recovery. The severity of ipsilateral *CST* injury was closely associated with the activity of daily living impairment in ICH patients.^7^ Our previous work had firstly revealed that striatum hemorrhage induced progressive destruction of the ultrastructure integrity of the *CST* at the cervical enlargement, which could prolong for at least 5 weeks.^8^

Accumulating evidence implicates intestinal bacteria is a crucial factor for the bidirectional communication between the gut and central nervous system.^9^ Ischemic stroke patients and transient ischemic attack patients displayed an obvious dysbiotic microbiota, characterized by lower abundance of beneficial commensal taxa and higher abundance of opportunistic pathogens.^10^ In an animal model, ICH caused significant gut dysbiosis and gastrointestinal function impairment, and bacteriotherapy by transplanting with healthy microbiota improved neurological function recovery by modulating immune response.^11^ The nucleotide‐binding oligomerization domain‐, leucine‐rich repeat and pyrin domain‐containing 3 (NLRP3) inflammasome is a molecular complex of the innate immune system and considered as a key contributor to the development and progression of a variety of neuropsychiatric disorders.^12^, ^13^ A growing body of research demonstrates that NLRP3 inflammasome plays an important role in the pathophysiology of ICH.^14^, ^15^, ^16^ In our previous study, selectively inhibiting NLRP3 inflammasome was sufficient to modulate the gut microbial composition, ameliorate *CST* injury of the cervical enlargement, and alleviate neurological deficits after ICH.^17^ Therefore, gut microbiota may be an important therapeutic target for the progression of ICH. However, the study on the signature of gut microbiota after ICH is still insufficient.

Oxymatrine (OMT) is a main quinolizidine alkaloid extracted from the traditional Chinese herb Sophorae Flavescentis Radix. Emerging studies demonstrated that OMT conferred neuroprotective effects on several neurological disorders, such as ischemic stroke and spinal cord injury.^18^, ^19^, ^20^ In preclinical models, OMT protected against cerebral ischemia–reperfusion injury by inhibiting pro‐inflammatory signaling pathways.^18^ It was reported that OMT could inhibit the progression of acute spinal cord injury by modulating inflammation, oxidative stress, and apoptosis through the TLR4/NF‐κB pathway.^19^ However, whether OMT exerted therapeutic effects on WMI after ICH, and the role of gut microbiota in these processes are largely unknown.

Therefore, the present study aimed to investigate whether OMT could attenuate hemorrhage‐induced WMI, especially secondary *CST* injury in cervical enlargement, and the role of gut microbiota in the underlying mechanism in an ICH model. We hypothesized that OMT alleviated ICH‐induced WMI and neurobehavioral deficits by modulating gut microbiota.

## 2 METHODS

### 2.1 Ethical approval

The experimental protocols were approved by the Institutional Ethics Committee of Zhujiang Hospital of Southern Medical University (LAEC‐2020‐227) and conducted according to the guidelines of the National Institute of Health Laboratory Animal Care and Use Guidelines.

### 2.2 Drug preparation and administration

**FIGURE 1:**
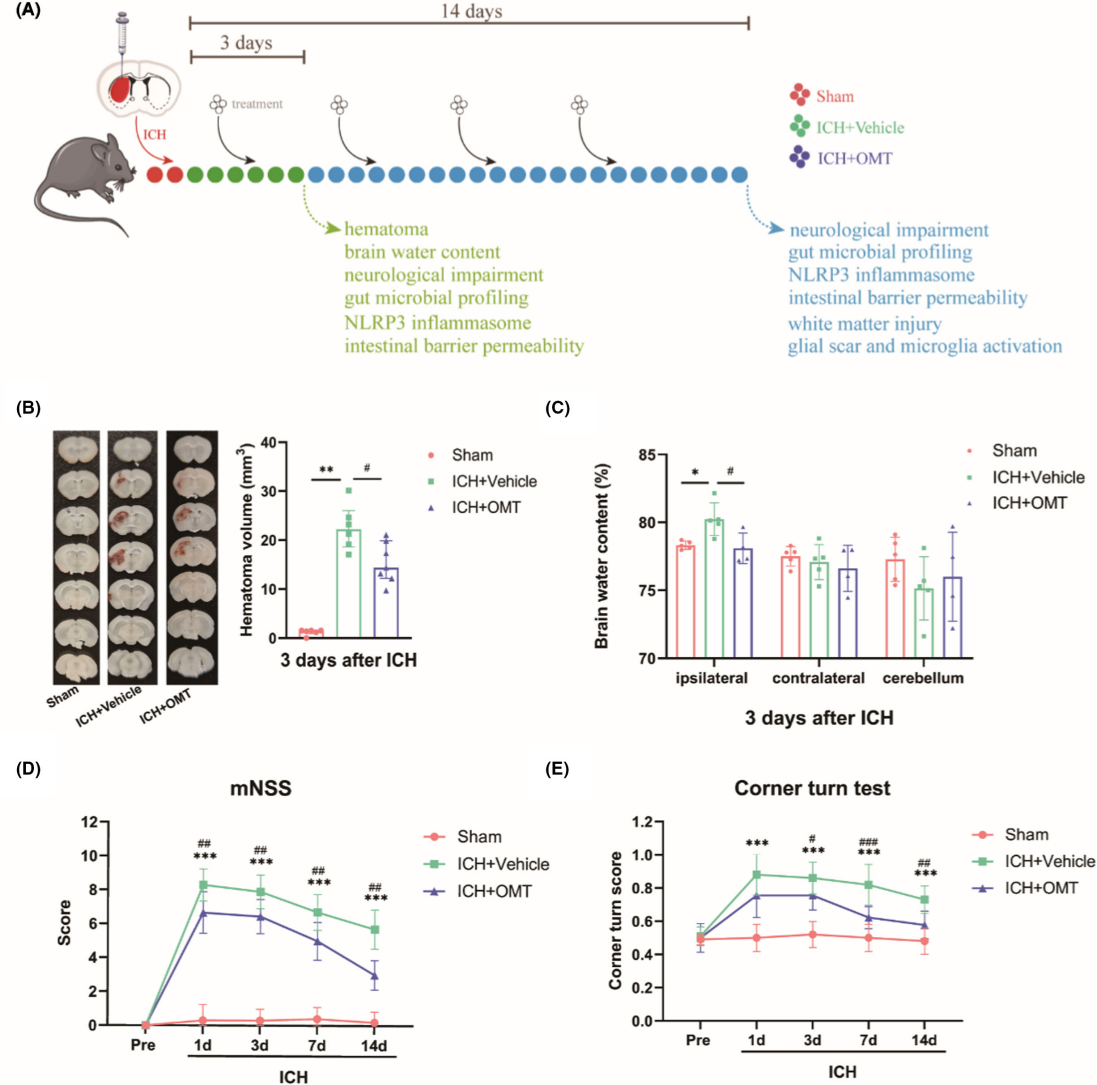
OMT alleviated the neurological deficits after ICH. (A) The experimental protocol for OMT treatment after ICH. (B, C) hematoma volume (B, *n* = 6–7/group) and brain water content (%) (C, *n* = 4‐5/group) at 3 days after ICH. (D, E) mNSS score (D, *n* = 9–10/group) and corner turn test (E, *n* = 9–10/group) at Days 1, 3, 7, and 14 after ICH. Sham group versus ICH + Vehicle group, *p<0.05, **p<0.01, ***p<0.001, ICH + Vehicle group versus ICH + OMT group, ^#^ *p*<0.05, ^##^ *p*<0.01, ^###^ *p*<0.001.

Animals were randomly assigned into three groups, namely Sham, ICH + Vehicle, and ICH + OMT group (Figure 1A). The mice of the ICH + OMT group daily received OMT (Cat#: A111286, Aladdin) by oral gavage, which was dissolved in deionized water to reach the concentration of 40 mg/ml, at 120 mg/kg body weight according to previous research,^18^ until sacrificed. Both the Sham group and the ICH + Vehicle group were given equivalent volumes of deionized water in the same manner.

### 2.3 Statistical analysis

Statistical analysis was performed using the SPSS software version 20.0 (SPSS). Normality was evaluated using Shapiro–Wilk's test. For normal distribution variables, the student *t*‐test was used to compare the means of two groups. Comparisons among multiple groups were analyzed using one‐way analysis of variance (ANOVA) with a post hoc analysis with the Bonferroni method. Continuous neurological function scores were analyzed by two‐way ANOVA adjusted by Tukey's post hoc test. Regarding non‐normal distribution variables, the Kruskal–Wallis H‐test and Mann–Whitney *U*‐test were conducted for statistical analysis between different groups. Correlation analysis between bacterial species and inflammatory markers was evaluated by Spearman's rank correlation. GraphPad Prism Software version 6.0 (GraphPad Prism) was applied to visualize analysis results. The *p*‐Value <0.05 was considered statistically significant. Other materials and methods are described in the Appendix S1.

## 3 RESULTS

### 3.1 OMT alleviated the neurological deficits after ICH

OMT by oral administration for 3 days reduced hematoma volume and brain water content (BWC, in ipsilateral hemilateral) compared with that of vehicle‐treated mice after ICH (Figure 1B,C). Then, we investigated inflammation by qPCR and found that NLRP3 inflammasome complex (Nlrp3, Asc, and Caspase‐1) and pro‐inflammatory cytokines (Il‐1β, Il‐6, Tnf‐α, and Nos2) mRNA levels were significantly elevated in peri‐hematoma region on Days 3 and 14, which were down‐regulated by OMT treatment (except Il‐6 at 3 days and Tnf‐α at 14 days) (Figure S1A,B).

The mNSS test showed that neurological function impairment reached the peak on Day 1 and thereafter gradually declined from Day 3 after ICH. Instead, OMT partially alleviated ICH‐induced neurological deficits (Figure 1D). Similarly, OMT administration significantly reduced the proportion of right turn after ICH (Figure 1E). Taken together, these results suggested that OMT could suppress hematoma expansion and brain edema development, inhibit neuroinflammation, alleviate neurological deficits.

### 3.2 OMT alleviated white matter injury and glial scar formation after ICH

**FIGURE 2:**
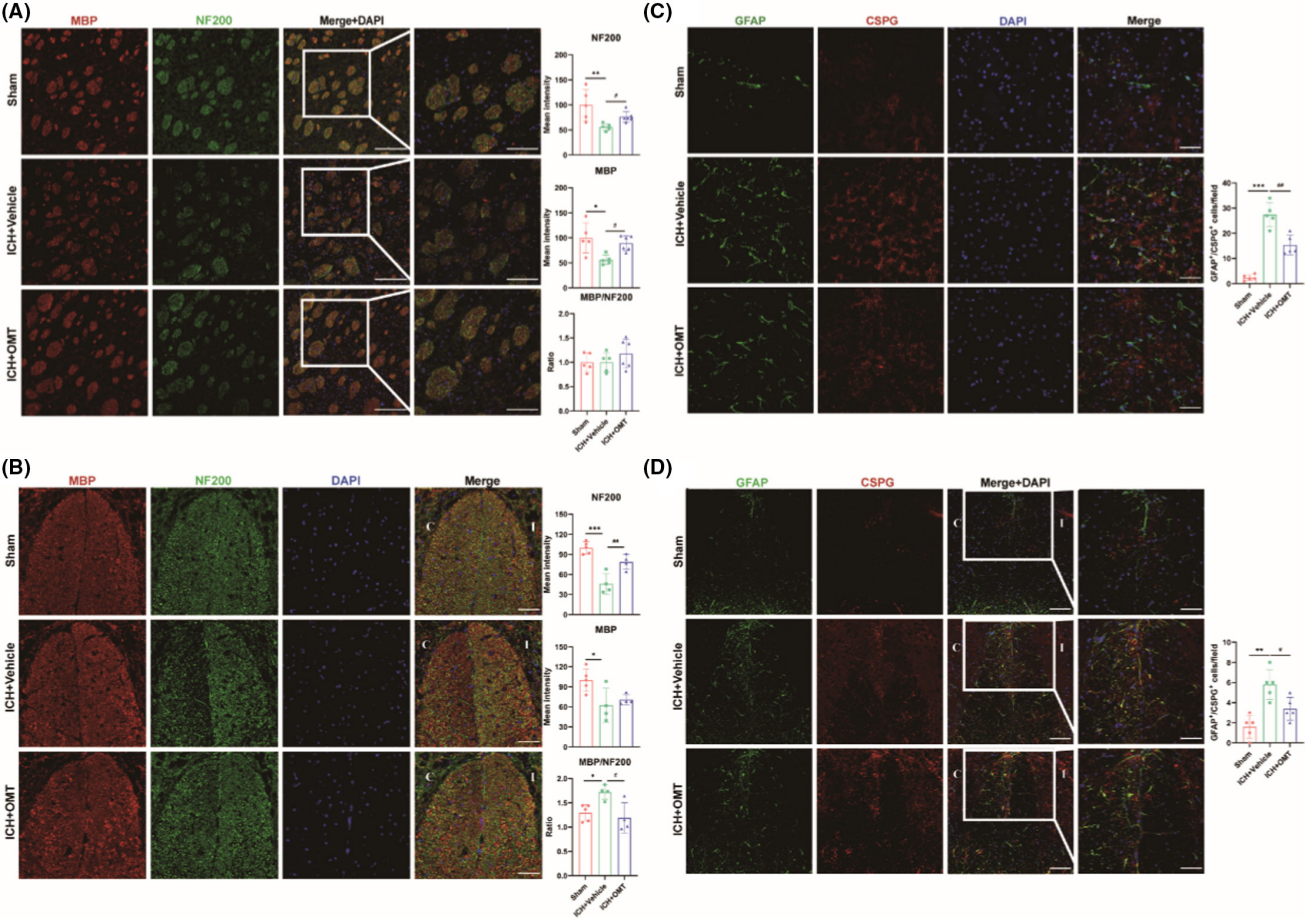
OMT alleviated white matter injury and the formation of glial scar after ICH. (A, B) Immunodetection for MBP and NF200 in the peri‐hematoma region (A) and distal CST injury region of cervical enlargement (B) at 14 days after ICH, and the result of quantitative analysis of their mean fluorescence intensity (MFI). The immunofluorescence amplification of representative images in the third column (A) was 10 × 20, Scale bar = 100 μm. The images in the fourth column are 10 × 40, Scale bar = 50 μm. *n* = 5‐6/group. The magnification of the immunofluorescence field was 10 × 40, Scale bar = 50 μm. *n* = 4‐5/group. (C, D) The expression of CSPG and GFAP in the peri‐hematoma region (C) and distal CST injury region of cervical enlargement (D) at 14 days after ICH. The magnification of the immunofluorescence field (C) was 10 × 40, Scale bar = 50 μm. The immunofluorescence amplification of images (D) was 10 × 20, Scale bar = 100 μm. The images within the white box in the last column are 10 × 40, Scale bar = 50 μm. Sham group versus ICH + Vehicle group, *p<0.05, **p<0.01, ***p<0.001, ICH + Vehicle group versus ICH + OMT group, ^#^ *p*<0.05, ^##^ *p*<0.01, ^###^ *p*<0.001.

The integrity of white matter was assessed by immunostaining with MBP (a marker of myelin sheath) and NF200 (a marker of axons) (Figure S2A). Our results demonstrated that both MBP and NF200 were markedly destroyed by ICH, resulting in sharp decrease in the mean fluorescence intensity (MFI) of MBP and NF200. The MFI of them was significantly increased in the OMT‐treated group (Figure 2A, Figure S2A). These findings suggested that OMT attenuated the WMI around the hematoma region.

Subsequently, we detected the expression of MBP and NF200 in the *CST* region of cervical enlargement to further clarify secondary WMI. The results showed that the expression of NF200 was dramatically decreased after ICH, consistent with that in the peri‐hematoma region, which indicated axonal Wallerian degeneration may extended to the *CST* region of cervical enlargement. The MFI of NF200 was significantly increased after OMT administration (Figure 2B). Nevertheless, the MFI of MBP in cervical enlargement was not significantly different in ICH mice with or without OMT treatment (Figure 2B). This may attribute to the heterogeneous pathogenic processes of the axonal degeneration and demyelination. Our results indicated that OMT attenuated both proximal and distal WMI at 14 days after ICH.

The formation of glial scar is regarded as another injury indicator after ICH. The chondroitin sulfate proteoglycan (CSPG) is an extracellular matrix compound, derived from reactive astrocytes, which is the major inhibitor of axonal growth. A mass of activated astrocytes, detected by the colocalization of GFAP and CSPG, aggregated at the peri‐hematoma region after ICH (Figure 2C, Figure S2B,C). The same phenomenon was observed in the *CST* region (Figure 2D, Figure S2D), suggesting similar pathogenic changes emerged in the distal WM of the spinal cord. Besides, ICH induced more activated microglia to aggregate in the *CST* region in response to axon injury and myelin destruction. While OMT treatment significantly attenuated glial scar formation and microgliosis in *CST* region (Figure 2C,D; Figure S2B–E). Our results indicated that OMT significantly inhibited the formation of glial scar and microgliosis at both proximal and distal WM after ICH.

### 3.3 OMT modulated the gut dysbiosis induced by ICH

**FIGURE 3:**
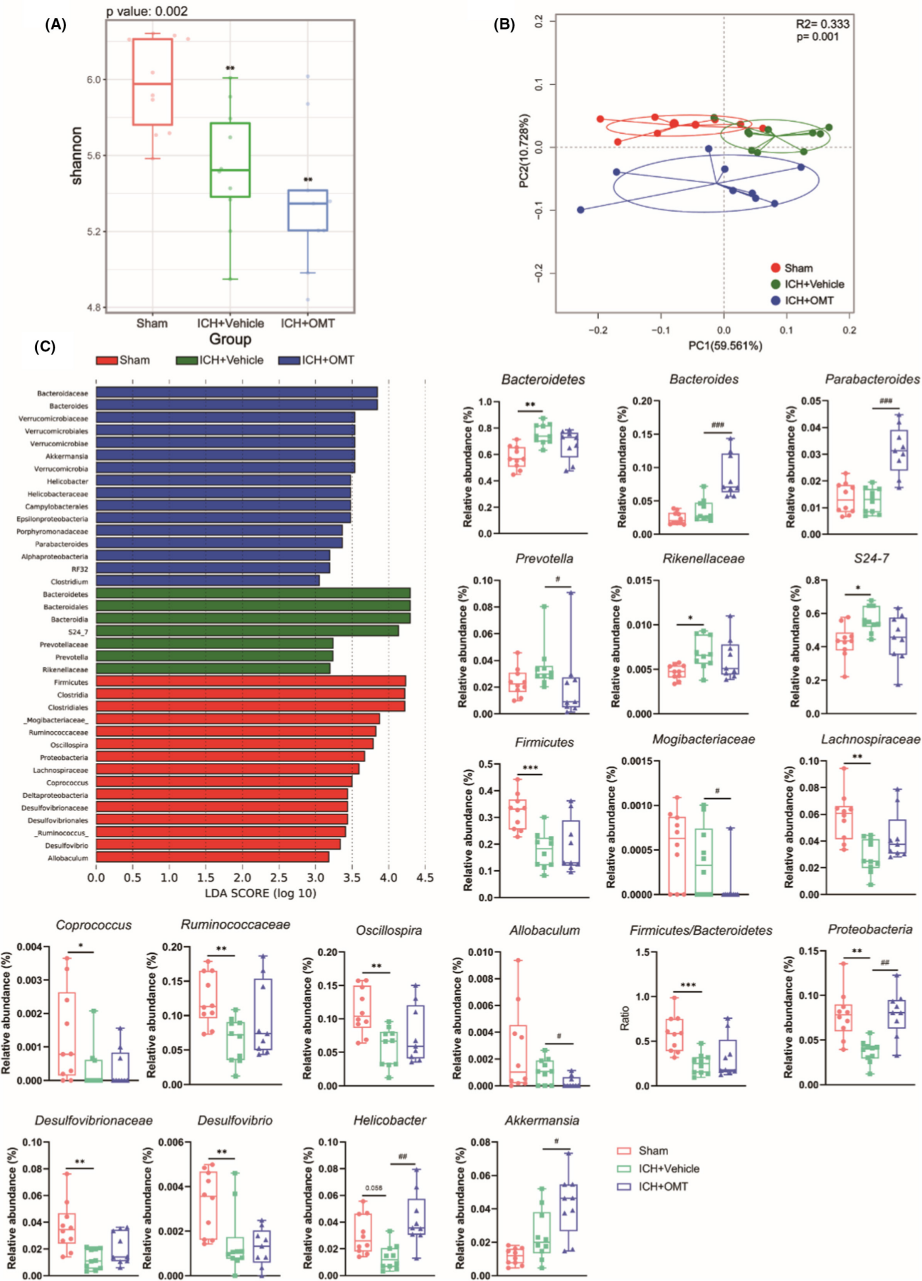
OMT modulated the gut dysbiosis at 3 days after ICH. (A‐B) Comparison of ɑ‐diversity (A, Shannon index) and β‐diversity (B, PCoA analysis based on weighted UniFrac distance) among three groups at 3 days after ICH. (C) The distribution bar plot based on the LEfSe analysis (LDA score (log 10)>3) and the richness of significantly differential taxa among three groups at 3 days after ICH. *n* = 9–10/group. Sham group versus ICH + Vehicle group, *p<0.05, **p<0.01, ***p<0.001, ICH + Vehicle group versus ICH + OMT group, ^#^ *p*<0.05, ^##^ *p*<0.01, ^###^ *p*<0.001.

To investigate the composition of gut microbiota after ICH and the effect of OMT on this alteration, 16 S rRNA gene sequencing was performed. Microbial profiling depicted that in the acute phase after ICH, the α‐diversity, calculated by the Shannon index, manifested a significant decline in both the vehicle‐ and OMT‐treated groups compared with the sham group (Figure 3A, Figure S3A). Additionally, the β‐diversity, reflecting the differences between microbial communities, estimated by PCoA analysis based on the weighted UniFrac distance, displayed that samples from the ICH + Vehicle group were clearly separated from those from the Sham group on Days 1 and 3 after ICH. And this phenomenon was only observed at 3 days after ICH between the ICH + Vehicle group and the ICH + OMT group (Figure 3B, Figure S3B). Microbial population analysis revealed that ICH remarkably altered the gut microbial composition, and OMT treatment modulated the composition of gut microbiota at the phyla and the genus levels. LEfSe analysis showed that at 1 day after ICH, the relative abundance of *Rikenellaceae* and *S24_7* was significantly higher but those of *Bacteroides*, *Parabacteroides*, *Coprococcus*, and *Proteobacteria* were significantly lower in the ICH + Vehicle group compared with the Sham group. While OMT significantly increased the relative abundance of *Bacteroides*, *Parabacteroides*, and *Proteobacteria* and decreased those of *Rikenellaceae* after ICH (Figure S3C–F). At 3 days after ICH, members from *Bacteroidetes*, *Rikenellaceae*, and *S24‐7* were excessively overgrown, while the relative abundances of *Lachnospiraceae*, *Coprococcus*, *Ruminococcaceae*, and *Oscillospira* which were mostly defined as butyrate‐producing bacteria, and other microbial species from *Firmicutes*, *Proteobacteria*, *Desulfovibrionaceae*, and *Desulfovibrio*, were all significantly decreased after ICH, and those were increased under OMT administration but with no statistical significance (except *Proteobacteria*), including the ratio of F/B. On the other hand, *Bacteroides*, *Parabacteroides*, *Helicobacter*, and *Akkermansia* were more enriched in the ICH + OMT group compared with the ICH + Vehicle group (Figure 3C, Figure S4A–C).

**FIGURE 4:**
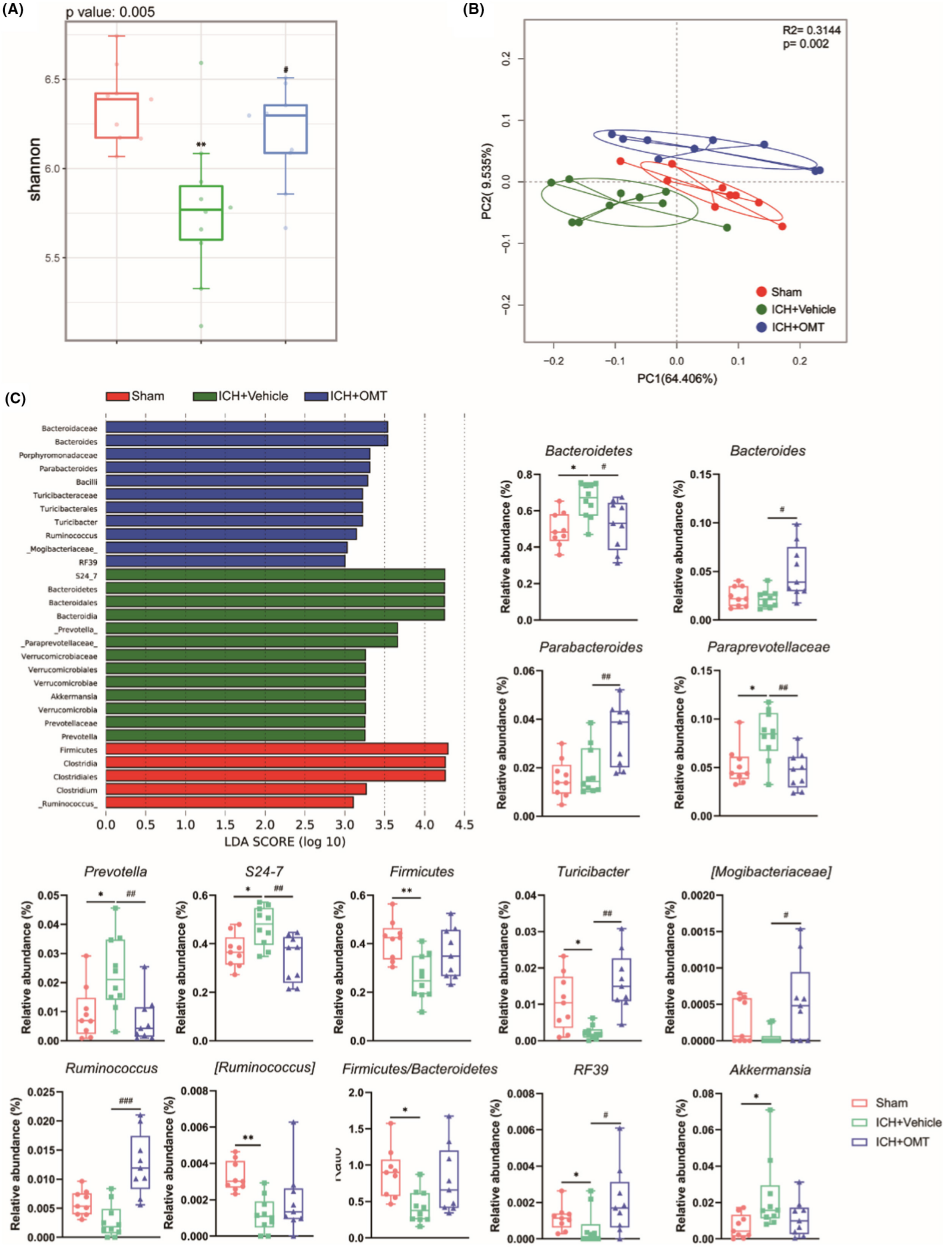
OMT modulated the gut dysbiosis at 14 days after ICH. (A, B) Comparison of ɑ‐diversity (A, Shannon index) and β‐diversity (B, PCoA analysis based on weighted UniFrac distance) among three groups at 14 days after ICH. (C) The distribution bar plot based on the LEfSe analysis (LDA score (log 10)>3) and the richness of significantly differential taxa among three groups at 14 days after ICH. *n* = 9–10/group. Sham group versus ICH + Vehicle group, *p<0.05, **p<0.01, ***p<0.001, ICH + Vehicle group versus ICH + OMT group, ^#^ *p*<0.05, ^##^ *p*<0.01, ^###^ *p*<0.001.

In the chronic phase after ICH, there was still a significant decline in the species richness in the ICH + Vehicle group as shown by the Shannon index (Figure 4A). PCoA analysis revealed that samples from different groups were clearly separated from each other, suggesting the composition of gut microbiota was discriminated between different groups (Figure 4B). Using LEfSe analysis, we determined that *Bacteroidetes*, *S24‐7*, *Paraprevotellaceae*, *Prevotella*, and *Akkermansia* become the predominated bacteria in the ICH + Vehicle group, but the relative abundance of above‐mentioned species (except *Akkermansia*) was significantly decreased in the ICH + OMT group (Figure 4C, Figure S4D–F). Meanwhile, ICH induced a lower abundance of *Firmicutes*, [*Ruminococcus*], and *Turicibacter*, and a lower F/B ratio. In contrast, *Bacteroides*, *Parabacteroides*, *Ruminococcus*, and *Turicibacter* significantly enriched in the ICH + OMT group compared with the ICH + Vehicle group (Figure 4C, Figure S4D–F). All these results indicated that ICH induced obvious gut dysbiosis, and OMT could modulate gut microbiota.

### 3.4 OMT ameliorated intestinal barrier disruption and gut‐derived endotoxemia after ICH

**FIGURE 5:**
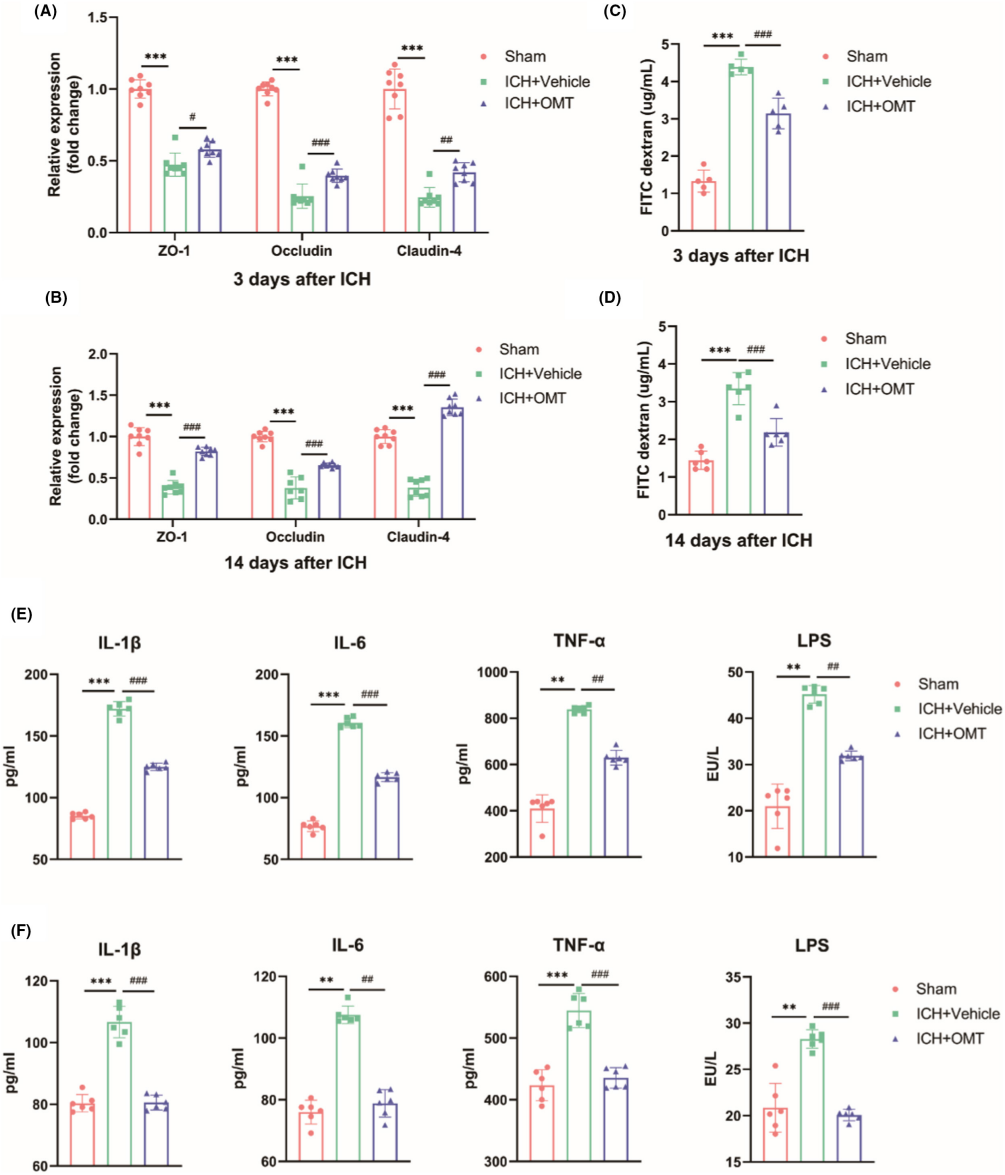
OMT ameliorated intestinal barrier disruption and gut‐derived endotoxemia after ICH. (A–D) The mRNA levels of tight junction proteins (ZO‐1, Occludin, and Claudin‐4. A, *n* = 8/group; B, *n* = 8/group) in colon tissues and serum FD4 concentrations (C, *n* = 5/group; D, *n* = 6/group) on Days 3 and 14 after ICH. (E, F) Serum LPS and inflammatory factors (IL‐1β, IL‐6, and TNF‐α) concentrations at 3d (E, *n* = 6/group) and 14d (F, *n* = 6/group) after ICH. (A) The heatmap indicated the correlation between microbial species and injury‐associated indexes on Days 3 (left) and 14 (right) after ICH. Red and green cells indicated positive and negative correlations, respectively. Sham group versus ICH + Vehicle group, *p<0.05, **p<0.01, ***p<0.001, ICH + Vehicle group versus ICH + OMT group, ^#^ *p*<0.05, ^##^ *p*<0.01, ^###^ *p*<0.001.

To determine intestinal barrier function, we detected the mRNA expression of intestinal tight junction proteins. The mRNA levels of target genes including ZO‐1, Occludin, and Claudin‐4 were significantly decreased on both Days 3 and 14 after ICH. Treatment with OMT significantly up‐regulated the expression levels of tight junction proteins (Figure 5A,B). Meanwhile, we evaluated the permeability of the intestinal barrier. The concentration of FD4 in the serum was increased after ICH at both time points, those level was decreased under OMT administration (Figure 5C,D). Taken together, ICH caused intestinal barrier dysfunction with tight junction proteins degradation and permeability increasing, while the application of OMT dramatically reversed the disruption of the intestinal barrier.

The serum concentrations of LPS and several inflammatory factors including TNF‐α, IL‐6, and IL‐1β were obviously increased after ICH, which were reversed by OMT (Figure 5E,F). In summary, OMT attenuated the gut‐derived endotoxemia after ICH.

### 3.5 The correlation between significantly differential microbiome taxa and injury‐associated indexes after ICH

**FIGURE 6:**
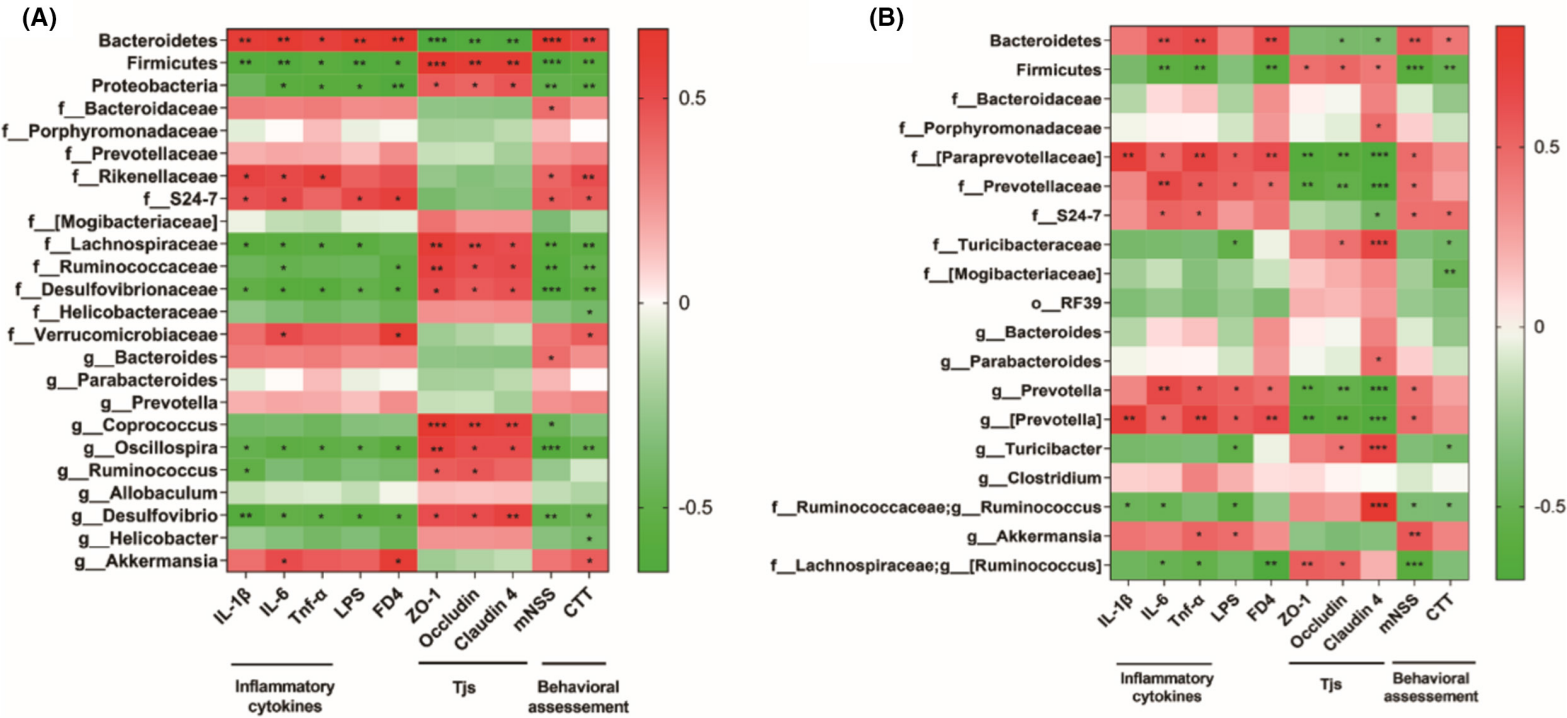
Correlation between significantly differential microbiome taxa and injury‐associated indexes after ICH. (A) The heatmap indicated the correlation between microbial species and injury‐associated indexes on Days 3 (left) and 14 (right) after ICH. Red and green cells indicated positive and negative correlations, respectively. *p<0.05, **p<0.01, ***p<0.001.

In order to investigate the correlation between the relative abundance of microbial species and injury‐associated indexes, Spearman's rank correlation was conducted. At 3 days after ICH, members from the *Bacteroidetes* phylum, including *Rikenellaceae* and *S24‐7*, were significantly positively correlated with several inflammatory markers (i.e., IL‐1β, IL‐6, TNF‐α, and LPS), serum concentration of FD4 and behavioral scores, but negatively correlated with the mRNA levels of intestinal tight junction proteins (Tjs). While there was a negative correlation between microbial species from *Firmicutes* and *Proteobacteria* phylum, specially *Lachnospiraceae*, *Desulfovibrionaceae*, *Ruminococcaceae*, *Oscillospira*, and *Desulfovibio* and those above markers except intestinal Tjs expression (Figure 6A). At 14 days after ICH, microbial species from *Bacteroidetes* phylum, such as *Paraprevotellaceae*, *Prevotellaceae S24‐7*, and *Prevotella*, still had a positive correlation with those markers, but negative correlation with Tjs expression. While a negative correlation was found between *Firmicutes*, *Ruminococcus*, and [*Ruminococcus*] and those markers, microbial taxa from *Firmicutes*, *Turicibacteraceae*, *Turicibacter*, *Ruminococcus*, and [*Ruminococcus*] had a significantly positive correlation with Tjs expression (Figure 6B). To sum up, the relative abundances of some microbial species were significantly associated with ICH‐induced pathological changes.

### 3.6 Gut microbiota partially mediated the therapeutic effects of OMT on ICH

**FIGURE 7:**
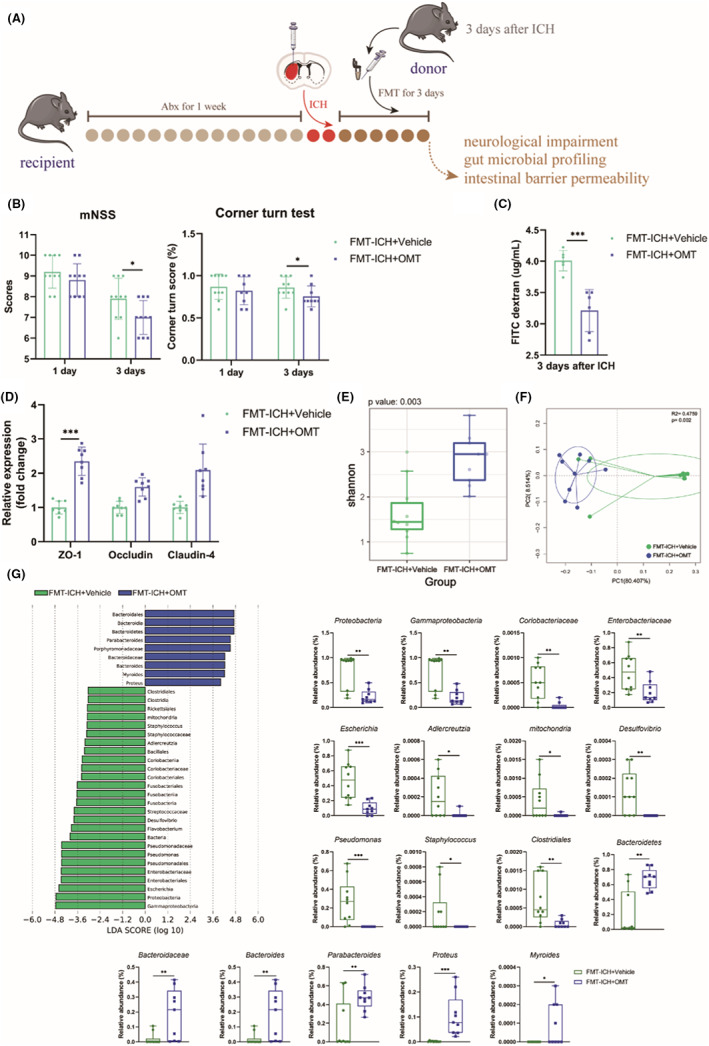
Gut microbiota mediated the therapeutic effects of OMT on ICH. (A) The experimental protocol for FMT after ICH. Donor mice, fecal samples were collected from ICH mice with or without OMT treatment on Day 3 after ICH. Recipient mice, C57BL/6J mice were subjected to antibiotics mix by gavage for 1 week, followed by ICH model conduction, and then daily received FMT for 3 days. (B) mNSS score (A, *n* = 10/group) and corner turn test (*n* = 9–10/group) on Days 1 and 3 after ICH. (C, D) Serum FD4 concentrations (C, *n* = 6/group) and the mRNA levels of tight junction proteins (D, ZO‐1, Occludin, and Claudin‐4, *n* = 8/group) in colon tissues on Day 3 after ICH. (E, F) Comparison of ɑ‐diversity (E, Shannon index), β‐diversity (F, PCoA analysis based on weighted UniFrac distance) at 3 days after ICH. (G) The distribution bar plot based on the LEfSe analysis (LDA score (log 10)>3), and the richness of significantly differential taxa between the two groups at 3 days after ICH. *n* = 9–10/group. Sham group versus ICH + Vehicle group, *p<0.05, **p<0.01, ***p<0.001, ICH + Vehicle group versus ICH + OMT group, ^#^ *p*<0.05, ^##^ *p*<0.01, ^###^ *p*<0.001.

We further conducted FMT to detect the role of the gut microbiota in the pathogenic processes of ICH (Figure 7A). At 3 days after ICH, recolonization with ICH + OMT‐fecal microbiota significantly reduced mNSS scores and right turn bias, compared with ICH‐fecal microbiota (Figure 7B). For intestinal barrier permeability detection, mice recolonized with ICH‐fecal microbiota exhibited higher serum fluorescein concentrations than those recolonized with ICH + OMT‐fecal microbiota, suggesting lower permeability of intestinal barrier after ICH + OMT‐fecal microbiota recolonization (Figure 7C). In addition, the mRNA levels of tight junction proteins were significantly increased in the FMT‐ICH + OMT group compared with the FMT‐ICH + Vehicle group (Figure 7D).

Furthermore, 16 S rRNA sequencing was performed to reveal the gut microbial composition in recipient mice who received FMT for 3 days. The α‐diversity in the FMT‐ICH + Vehicle group was significantly lower than those in the FMT‐ICH + OMT group (Figure 7E). As the PCoA analysis showed, the distribution of samples from the FMT‐ICH + Vehicle group was clearly separated from those from the FMT‐ICH + OMT group, and the difference in PC1 axis reached significant (Figure 7F). Population analysis demonstrated that the FMT‐ICH + Vehicle group displayed a higher abundance of *Proteobacteria* and a lower abundance of *Bacteroidetes* than the FMT‐ICH + OMT group at the phylum level (Figure S4G). The abundance of the predominant seven genus was different between the two groups at the genus level (Figure S4H). The result of LEfSe analysis revealed that members from the *Proteobacteria* phylum, such as *Coriobacteriaceae*, *Enterobacteriaceae*, *Escherichia*, *Adlercreutzia*, *mitochondria*, *Desulfovibrio*, *Pseudomonas*, *Staphylococcus*, and *Clostridiales*, were enriched in the FMT‐ICH + Vehicle group, compared to those in the FMT‐ICH + OMT group (Figure 7G, Figure S4I). While the relative abundance of *Bacteroidetes*, *Bacteroidaceae*, *Bacteroides*, *Parabacteroides*, *Proteus*, and *Myroides* in the FMT‐ICH + Vehicle group were significantly lower than those in the FMT‐ICH + OMT group (Figure 7G, Figure S4I). Above all, the therapeutic effects of OMT on ICH‐induced neurological deficits and intestinal barrier dysfunction were partially mediated by gut microbiota. However, the mechanism underlying requires a more thoroughly designed study to investigate.

## 4 DISCUSSION

WMI induced by ICH plays an important role in the neurological function outcome.^21^ In this study, it was shown that ICH can not only cause extensive WMI in the primary lesion but also induce persistent secondary white matter fiber tracts injury at the cervical enlargement, followed by gut dysbiosis, intestinal barrier dysfunction, and systemic inflammation. Oral administration with OMT significantly alleviated ICH‐induced WMI in the hematoma region and distal spinal segment, modulated gut microbial composition, and ameliorated intestinal barrier dysfunction. FMT experiment confirmed that gut microbiota was involved in this pathophysiological process after ICH.

Baseline hematoma volume and subsequently hematoma expansion are established prognostic factors of both mortality and functional outcome after ICH.^22^, ^23^ Besides, secondary brain injury caused by mass effect and release of blood components, such as edema and inflammation, also plays an important role in the neurological function recovery after ICH.^24^ Current study revealed that OMT administration (120 mg/kg) conferred neuroprotective effects on ICH by reducing hematoma volume and inhibiting the formation of edema. Accumulating studies demonstrated that OMT displayed remarkably antiinflammation and immunomodulation effects. NLRP3 inflammasome is a critical receptor and sensor of the innate immune system that aggravated inflammatory response and brain edema progression after ICH, and selectively inhibiting NLRP3 inflammasome activation attenuated ICH‐induced brain injury and neurological function deficits.^14^, ^25^, ^26^ Our study showed that OMT significantly downregulated NLRP3 inflammasome complex and other pro‐inflammatory cytokines mRNA levels at 3 and 14 days after ICH, indicating OMT could effectively inhibit neuroinflammation in the brain. However, the underlying mechanism of the inhibition effect of OMT on NLRP3 inflammasome and neuroinflammation still needs to be verified in further study.

The severity of WMI is closely related to neuromotor dysfunction in ICH patients.^7^ In present study, OMT significantly alleviated WMI in the peri‐hematoma region at 14 days after ICH. The less extensive WMI in the primary lesion may be account for better neurological function recovery after ICH. Besides, ICH‐induced secondary remote white matter fiber tracts (such as *CST*) injury also plays a pivotal role in the functional outcome. In stroke patients, the integrity of the *CST* was a determining factor for the proportional recovery of motor impairment.^27^ Quantitative tractography revealed that there were more reconstructed *CST* fiber pathways in ICH patients with favorable outcomes.^28^ Based on the results above, treatment with OMT significantly alleviated ICH‐induced *CST* axon injury and demyelination at cervical enlargement, which is perhaps due to the protection of OMT against WMI in the primary lesion, or its inhibition of astrocytes/microglia activation and inflammatory response. Prolonged activation of microglia induced by myelin debris has long been implicated in aggravating inflammatory action by secreting pro‐inflammatory cytokines.^29^, ^30^ Whereas Cunha et al. revealed that the pro‐inflammatory phagocytic phenotype of microglia is essential for myelin debris clearance and remyelination.^31^ In our study, microglia activation mitigated after OMT treatment perhaps due to the directed inhibition effect of OMT or less severe *CST* injury. Furthermore, OMT significantly alleviated ICH‐induced neurological deficits both in the acute phase and the chronic phase. Perhaps slighter primary WMI in the peri‐hematoma region and secondary *CST* injury account for better neurobehavioral performance after OMT treatment.

Gut microbiota is involved in the development and progression of ischemic stroke, through the microbiota–gut–brain axis.^32^ Recolonized with stroke‐induced dysbiotic microbiome from special pathogen‐free mice, germ‐free mice developed a larger infarct volume after distal middle cerebral artery occlusion.^33^ However, the role of gut microbiota in the ICH outcome is still poorly understood. Interestingly, when administrated orally, OMT will be converted into a more absorbable metabolite matrine (MT) by gut bacteria in the gastrointestinal tract.^34^ 16 S rRNA sequencing revealed that oral administration with MT dramatically sharped gut microbial community, resulting in more beneficial commensal genera, such as *Ruminoclostridium*, *Lachnospiraceae*, and *Ruminococcaceae*.^35^ Whether OMT regulated gut microbial composition remained largely unknown. Our results revealed that ICH caused persistent microbiome disturbances at consecutive time courses. OMT significantly modulated gut microbial composition after ICH. Moreover, correlation analysis revealed that bacteria richness were significantly correlated with inflammation, intestinal barrier permeability, and neurological deficits after ICH. However, little is known about the precise mechanism by which differential bacteria affected the pathological process of ICH.

Intestinal injury, especially the gastrointestinal bleeding, is a common critical complication in ICH patients and an important risk factor for poor outcomes.^36^, ^37^ In an animal model, ICH induced rapid and persistent impairment of intestinal barrier function and inflammation.^38^ Another study by Yu et al. also found that gastrointestinal dysfunction, with increasing intestinal barrier permeability, and disrupting intestinal integrity, occurred after ICH.^11^ In this study, ICH induced significant disruption of intestinal barrier integrity and increased intestinal permeability, inflammatory cytokines, and LPS levels in the bloodstream. OMT significantly reversed these pathological changes induced by ICH and significantly alleviated the impairment of intestinal function and circulating inflammatory response.

Although our study has provided evidence that oral administration of OMT significantly alleviated ICH‐induced neurological deficits, gut dysbiosis, intestinal barrier dysfunction, and systemic inflammation, and FMT confirmed that gut microbiota plays an important role in these pathological processes, there were still several limitations in the present study. Firstly, medicine metabolism by bacteria in the intestine usually affects their pharmacokinetics and efficacy.^39^, ^40^, ^41^ Further research investigating the role of OMT metabolite by the microbiome in ICH is warranted. Secondly, the impact of OMT on intestinal metabolites derived from the microbiome requires further study. Thirdly, although the role of gut microbiota in ICH was verified by the FMT experiment, the underlying mechanism still needs further study to elucidate.

## 5 CONCLUSIONS

In conclusion, our study is the first time to reveal that OMT treatment effectively reduced WMI both at the striatum and distal *CST* region of the cervical enlargement, improved ICH‐caused gut dysbiosis, promoted the intestinal barrier reconstruction, and inhibited the systemic inflammatory response. The role of intestinal flora in the effects of OMT on alleviating WMI induced by ICH still needs to be further studied.

## AUTHOR CONTRIBUTIONS

The work presented here was carried out in collaboration among all authors. Haitao Sun conceived and designed the study. Hongwei Zhou, Wen Yuan, and Junshan Liu provided support of essential laboratory resources for this study. Jing Li conducted this study and drafted this manuscript; Jianhao Liang, Meiqin Zeng, Kaijian Sun, Yunhao Luo, Huaping Zheng, and Feng Li analyzed the data. Haitao Sun revised the manuscript. All authors reviewed and approved the final manuscript.

## FUNDING INFORMATION

This work was supported by Basic and Applied Basic Research Foundation of Guangdong Province (2020A1515010038), Key‐Area Research and Development Program of Guangdong Province (2018B030340001), Presidential Foundation of Zhujiang Hospital, Southern Medical University (yzjj2018rc03), Guangdong Province Universities and Colleges Pearl River Scholar Funded Scheme (GDHVPS2018), Young Elite Scientists Sponsorship Program by CACM (2019‐QNRC2‐C14), and Scientific research project funded by health Commission of Guangxi Zhuang Autonomous Region, China (Z20211397).

## CONFLICT OF INTEREST

The authors declare no conflict of interest.

## CONSENT FOR PUBLICATION

Not applicable.

## Supporting information


Figure S1.

Figure S2.

Figure S3.

Figure S4.

Appendix S1.

## Data Availability

All raw data used in this manuscript are available from the corresponding author upon reasonable request.

## References

[1] Global, regional, and national burden of stroke and its risk factors, 1990‐2019: a systematic analysis for the global burden of disease study 2019. Lancet Neurol. 2021;20(10):795‐820. 34487721 10.1016/S1474-4422(21)00252-0 PMC8443449

[2] Magid‐Bernstein J , Girard R , Polster S , et al. Cerebral hemorrhage: pathophysiology, treatment, and future directions. Circ Res. 2022;130(8):1204‐1229. 35420918 10.1161/CIRCRESAHA.121.319949 PMC10032582

[3] Liu Y , Xia Y , Wang X , et al. White matter hyperintensities induce distal deficits in the connected fibers. Hum Brain Mapp. 2021;42(6):1910‐1919. 33417309 10.1002/hbm.25338 PMC7978134

[4] Yu X , Jiaerken Y , Wang S , et al. Changes in the corticospinal tract beyond the ischemic lesion following acute hemispheric stroke: a diffusion kurtosis imaging study. J Magn Reson Imaging. 2020;52(2):512‐519. 31981400 10.1002/jmri.27066

[5] Smith EE , Gurol ME , Eng JA , et al. White matter lesions, cognition, and recurrent hemorrhage in lobar intracerebral hemorrhage. Neurology. 2004;63(9):1606‐1612. 15534243 10.1212/01.wnl.0000142966.22886.20

[6] Zhai F , Liu J , Su N , et al. Disrupted white matter integrity and network connectivity are related to poor motor performance. Sci Rep. 2020;10(1):18369. 33110225 10.1038/s41598-020-75617-1 PMC7591496

[7] Jang SH , Choi EB . Relation between the corticospinal tract state and activities of daily living in patients with intracerebral hemorrhage. Stroke. 2022;53(2):544‐551. 34538084 10.1161/STROKEAHA.121.034939 PMC8785518

[8] Ng ACK , Yao M , Cheng SY , et al. Protracted morphological changes in the corticospinal tract within the cervical spinal cord after intracerebral hemorrhage in the right striatum of mice. Front Neurosci. 2020;14:506. 32581678 10.3389/fnins.2020.00506 PMC7290159

[9] Cryan JF , O'Riordan KJ , Cowan CSM , et al. The microbiota‐gut‐brain Axis. Physiol Rev. 2019;99(4):1877‐2013. 31460832 10.1152/physrev.00018.2018

[10] Yin J , Liao SX , He Y , et al. Dysbiosis of gut microbiota with reduced trimethylamine‐N‐oxide level in patients with large‐artery atherosclerotic stroke or transient ischemic attack. J Am Heart Assoc. 2015;4(11):e002699. 26597155 10.1161/JAHA.115.002699 PMC4845212

[11] Yu X , Zhou G , Shao B , et al. Gut microbiota dysbiosis induced by intracerebral hemorrhage aggravates neuroinflammation in mice. Front Microbiol. 2021;12:647304. 34025607 10.3389/fmicb.2021.647304 PMC8137318

[12] Walsh JG , Muruve DA , Power C . Inflammasomes in the CNS. Nat Rev Neurosci. 2014;15(2):84‐97. 24399084 10.1038/nrn3638

[13] Swanson KV , Deng M , Ting JP . The NLRP3 inflammasome: molecular activation and regulation to therapeutics. Nat Rev Immunol. 2019;19(8):477‐489. 31036962 10.1038/s41577-019-0165-0 PMC7807242

[14] Ma Q , Chen S , Hu Q , Feng H , Zhang JH , Tang J . NLRP3 inflammasome contributes to inflammation after intracerebral hemorrhage. Ann Neurol. 2014;75(2):209‐219. 24273204 10.1002/ana.24070 PMC4386653

[15] Ren H , Kong Y , Liu Z , et al. Selective NLRP3 (pyrin domain‐containing protein 3) inflammasome inhibitor reduces brain injury after intracerebral hemorrhage. Stroke. 2018;49(1):184‐192. 29212744 10.1161/STROKEAHA.117.018904 PMC5753818

[16] Zhang Z , Guo P , Huang S , et al. Inhibiting microglia‐derived NLRP3 alleviates subependymal edema and cognitive dysfunction in posthemorrhagic hydrocephalus after intracerebral hemorrhage via AMPK/Beclin‐1 pathway. Oxid Med Cell Longev. 2022;2022:4177317. 35620574 10.1155/2022/4177317 PMC9129981

[17] Xiao L , Zheng H , Li J , et al. Targeting NLRP3 inflammasome modulates gut microbiota, attenuates corticospinal tract injury and ameliorates neurobehavioral deficits after intracerebral hemorrhage in mice. Biomed Pharmacother. 2022;149:112797. 35279596 10.1016/j.biopha.2022.112797

[18] Liu Y , Zhang XJ , Yang CH , Fan HG . Oxymatrine protects rat brains against permanent focal ischemia and downregulates NF‐kappaB expression. Brain Res. 2009;1268:174‐180. 19285049 10.1016/j.brainres.2009.02.069

[19] Guan B , Chen R , Zhong M , Liu N , Chen Q . Protective effect of oxymatrine against acute spinal cord injury in rats via modulating oxidative stress, inflammation and apoptosis. Metab Brain Dis. 2020;35(1):149‐157. 31840202 10.1007/s11011-019-00528-8

[20] Zhou S , Qiao B , Chu X , Kong Q . Oxymatrine attenuates cognitive deficits through SIRT1‐mediated autophagy in ischemic stroke. J Neuroimmunol. 2018;323:136‐142. 30196826 10.1016/j.jneuroim.2018.06.018

[21] Fu X , Zhou G , Zhuang J , et al. White matter injury after intracerebral hemorrhage. Front Neurol. 2021;12:562090. 34177751 10.3389/fneur.2021.562090 PMC8222731

[22] Davis SM , Broderick J , Hennerici M , et al. Hematoma growth is a determinant of mortality and poor outcome after intracerebral hemorrhage. Neurology. 2006;66(8):1175‐1181. 16636233 10.1212/01.wnl.0000208408.98482.99

[23] Hemphill JC 3rd , Bonovich DC , Besmertis L , Manley GT , Johnston SC . The ICH score: a simple, reliable grading scale for intracerebral hemorrhage. Stroke. 2001;32(4):891‐897. 11283388 10.1161/01.str.32.4.891

[24] Keep RF , Hua Y , Xi G . Intracerebral haemorrhage: mechanisms of injury and therapeutic targets. Lancet Neurol. 2012;11(8):720‐731. 22698888 10.1016/S1474-4422(12)70104-7 PMC3884550

[25] Yuan B , Shen H , Lin L , Su T , Zhong S , Yang Z . Recombinant adenovirus encoding NLRP3 RNAi attenuate inflammation and brain injury after intracerebral hemorrhage. J Neuroimmunol. 2015;287:71‐75. 26439964 10.1016/j.jneuroim.2015.08.002

[26] Yao ST , Cao F , Chen JL , et al. NLRP3 is required for complement‐mediated Caspase‐1 and IL‐1beta activation in ICH. J Mol Neurosci. 2017;61(3):385‐395. 27933491 10.1007/s12031-016-0874-9

[27] Stinear CM , Byblow WD , Ackerley SJ , Smith MC , Borges VM , Barber PA . Proportional motor recovery after stroke: implications for trial design. Stroke. 2017;48(3):795‐798. 28143920 10.1161/STROKEAHA.116.016020

[28] Volbers B , Mennecke A , Kästle N , et al. Quantitative corticospinal tract assessment in acute intracerebral hemorrhage. Transl Stroke Res. 2021;12(4):540‐549. 32954472 10.1007/s12975-020-00850-9 PMC8213667

[29] Block ML , Zecca L , Hong JS . Microglia‐mediated neurotoxicity: uncovering the molecular mechanisms. Nat Rev Neurosci. 2007;8(1):57‐69. 17180163 10.1038/nrn2038

[30] Perry VH , Teeling J . Microglia and macrophages of the central nervous system: the contribution of microglia priming and systemic inflammation to chronic neurodegeneration. Semin Immunopathol. 2013;35(5):601‐612. 23732506 10.1007/s00281-013-0382-8 PMC3742955

[31] Cunha MI , Su M , Cantuti‐Castelvetri L , et al. Pro‐inflammatory activation following demyelination is required for myelin clearance and oligodendrogenesis. J Exp Med. 2020;217(5):e20191390. 32078678 10.1084/jem.20191390 PMC7201919

[32] Cryan JF , O'Riordan KJ , Sandhu K , Peterson V , Dinan TG . The gut microbiome in neurological disorders. Lancet Neurol. 2020;19(2):179‐194. 31753762 10.1016/S1474-4422(19)30356-4

[33] Singh V , Roth S , Llovera G , et al. Microbiota dysbiosis controls the neuroinflammatory response after stroke. J Neurosci. 2016;36(28):7428‐7440. 27413153 10.1523/JNEUROSCI.1114-16.2016 PMC6705544

[34] Gu Y , Lu J , Sun W , et al. Oxymatrine and its metabolite matrine contribute to the hepatotoxicity induced by radix Sophorae tonkinensis in mice. Exp Ther Med. 2019;17(4):2519‐2528. 30906440 10.3892/etm.2019.7237 PMC6425122

[35] Wu H , Chen Q , Liu J , et al. Microbiome analysis reveals gut microbiota alteration in mice with the effect of matrine. Microb Pathog. 2021;156:104926. 33964419 10.1016/j.micpath.2021.104926

[36] Yang TC , Li JG , Shi HM , et al. Gastrointestinal bleeding after intracerebral hemorrhage: a retrospective review of 808 cases. Am J Med Sci. 2013;346(4):279‐282. 23221511 10.1097/MAJ.0b013e318271a621

[37] Wang WJ , Lu JJ , Wang YJ , et al. Clinical characteristics, management, and functional outcomes in Chinese patients within the first year after intracerebral hemorrhage: analysis from China National Stroke Registry. CNS Neurosci Ther. 2012;18(9):773‐780. 22943144 10.1111/j.1755-5949.2012.00367.x PMC6493640

[38] Cheng Y , Zan J , Song Y , Yang G , Shang H , Zhao W . Evaluation of intestinal injury, inflammatory response and oxidative stress following intracerebral hemorrhage in mice. Int J Mol Med. 2018;42(4):2120‐2128. 30015849 10.3892/ijmm.2018.3755

[39] Koppel N , Maini Rekdal V , Balskus EP . Chemical transformation of xenobiotics by the human gut microbiota. Science. 2017;356(6344):eaag2770. 28642381 10.1126/science.aag2770 PMC5534341

[40] Javdan B , Lopez JG , Chankhamjon P , et al. Personalized mapping of drug metabolism by the human gut microbiome. Cell. 2020;181(7):1661‐1679.e1622. 32526207 10.1016/j.cell.2020.05.001 PMC8591631

[41] McCoubrey LE , Gaisford S , Orlu M , Basit AW . Predicting drug‐microbiome interactions with machine learning. Biotechnol Adv. 2022;54:107797. 34260950 10.1016/j.biotechadv.2021.107797

