# Supplementary figures and images for: Oxymatrine ameliorates white matter injury by modulating gut microbiota after intracerebral hemorrhage in mice
Source: CNS Neurosci Ther. 2022 Dec 22;29(Suppl 1):18–30. doi: 10.1111/cns.14066 (PMC10314101; doi:10.1111/cns.14066)

FIGURE S1


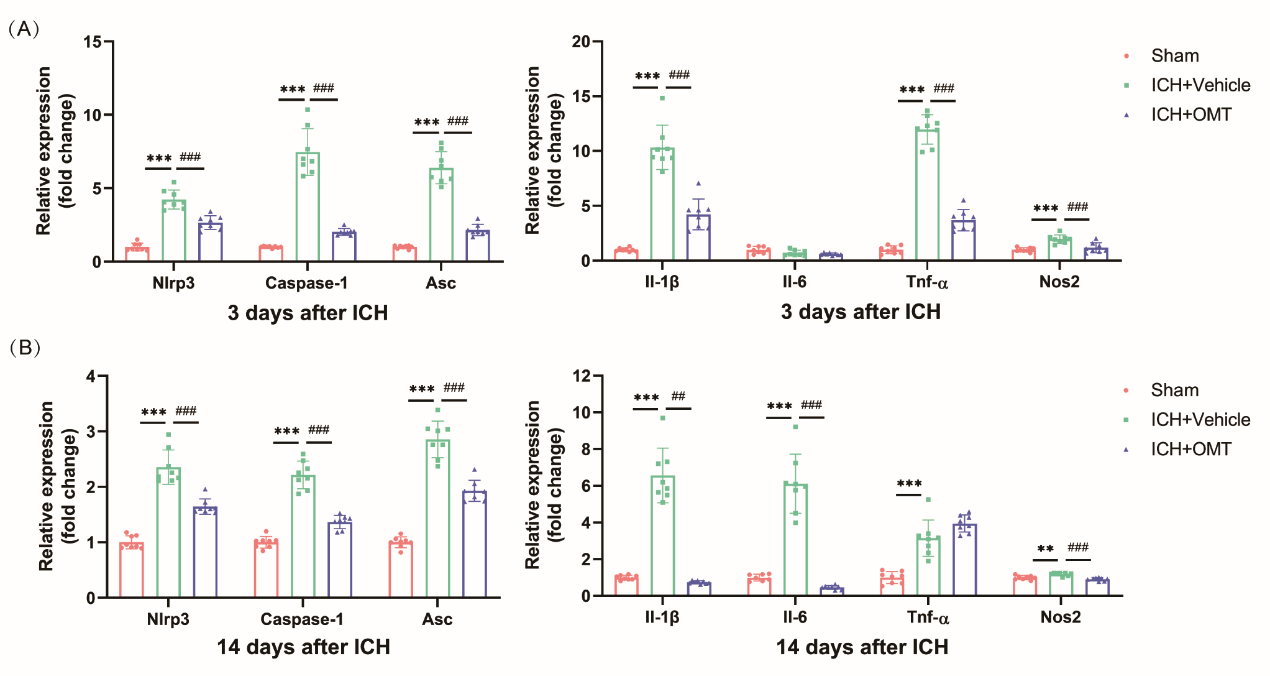

Supplement: Supplementary file 1 — Figure S1. [file CNS-29-18-s002.docx]

FIGURE S2


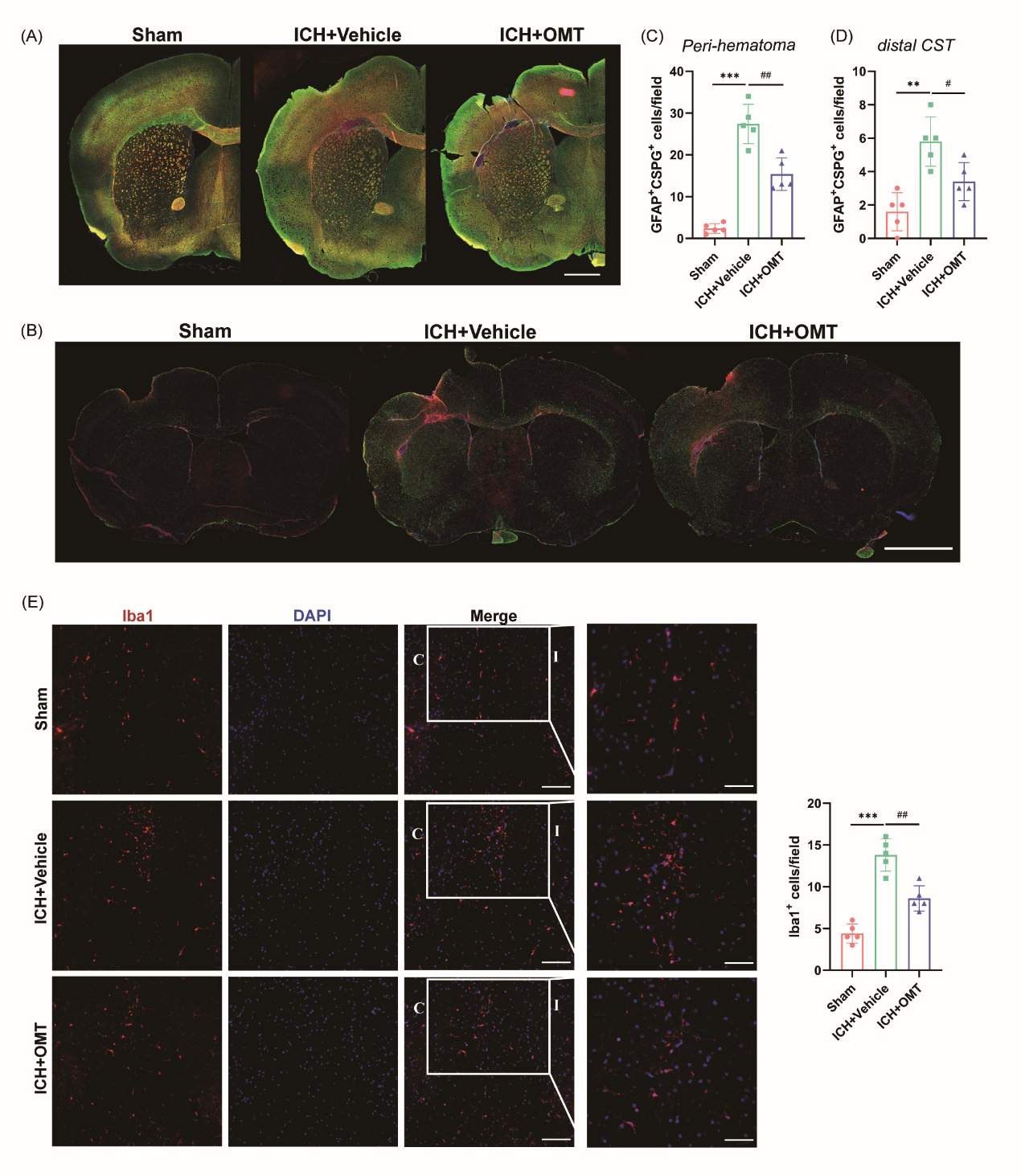

Supplement: Supplementary file 2 — Figure S2. [file CNS-29-18-s004.docx]

FIGURE S3


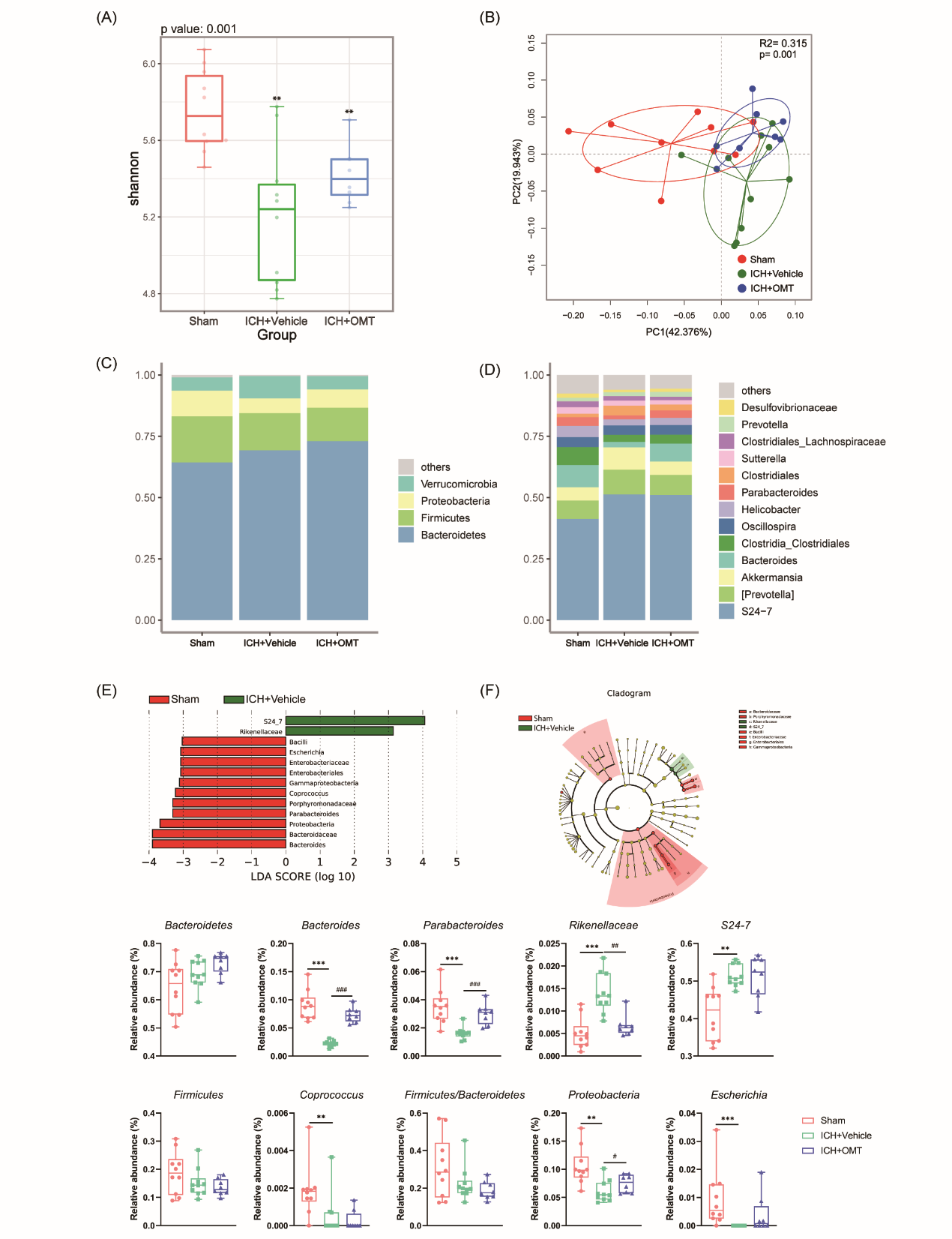

Supplement: Supplementary file 3 — Figure S3. [file CNS-29-18-s005.docx]

FIGURE S4


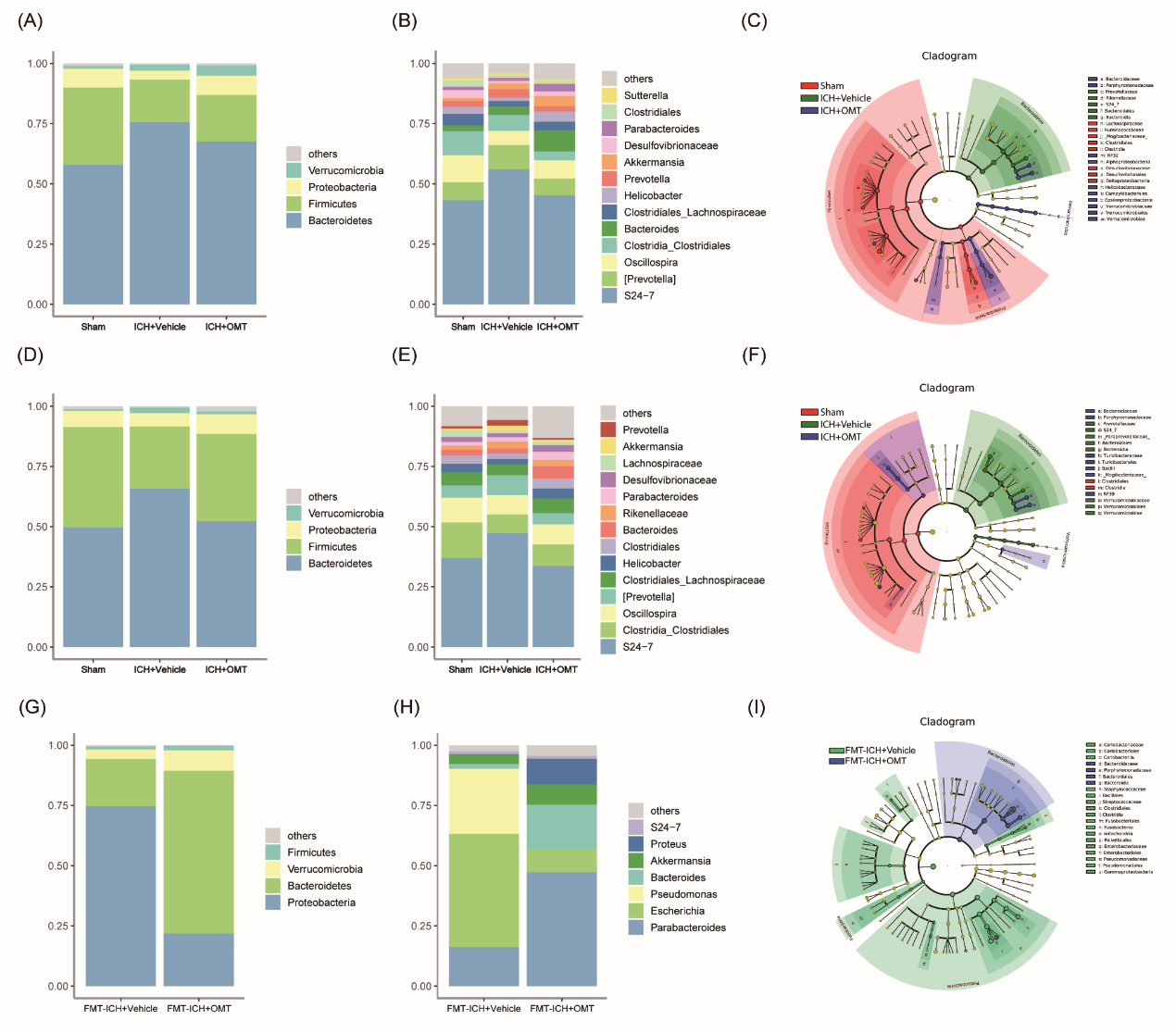

Supplement: Supplementary file 4 — Figure S4. [file CNS-29-18-s001.docx]
