# Supplementary material for: Oxymatrine ameliorates white matter injury by modulating gut microbiota after intracerebral hemorrhage in mice
Source: CNS Neurosci Ther. 2022 Dec 22;29(Suppl 1):18–30. doi: 10.1111/cns.14066 (PMC10314101; doi:10.1111/cns.14066)
Supplement: Supplementary file 5 — Appendix S1. [file CNS-29-18-s003.docx]

**Oxymatrine ameliorates white matter injury by modulating gut microbiota after intracerebral hemorrhage in mice**

Jing Li^1,2^, Jianhao Liang^1^, Meiqin Zeng^1^, Kaijian Sun^1^, Yunhao Luo^1^, Huaping Zheng^2^, Feng Li^2^, Wen Yuan^3^, Hongwei Zhou^1^, Junshan Liu^4^, Haitao Sun^1, 2, 5*^

^1^ Clinical Biobank Center, Microbiome Medicine Center, Department of Laboratory Medicine, Zhujiang Hospital, Southern Medical University, Guangzhou 510280, China

^2^ Neurosurgery Center, The National Key Clinical Specialty, The Engineering Technology Research Center of Education Ministry of China on Diagnosis and Treatment of Cerebrovascular Disease, Guangdong Provincial Key Laboratory on Brain Function Repair and Regeneration, The Neurosurgery Institute of Guangdong Province, Zhujiang Hospital, Southern Medical University, Guangzhou 510280, China

^3^ Laboratory Animal Center, Zhujiang Hospital, Southern Medical University, Guangzhou 510280, China

^4^ Department of Pharmacology of Chinese Medicine, School of Traditional Chinese Medicine, Southern Medical University, Guangzhou 510515, China

^5^ Key Laboratory of Mental Health of the Ministry of Education, Guangdong-Hong Kong-Macao Greater Bay Area Centre for Brain Science and BrainInspired Intelligence, Southern Medical University, Guangzhou 510515, China

* Corresponding author:

Haitao Sun: [2009sht@smu.edu.cn](mailto:2009sht@smu.edu.cn)

**METHODS**

**Model of intracerebral hemorrhage**

ICH model was conducted as previously described^1^. Briefly, anesthesia was performed by intraperitoneal injection of tribromoethanol (0.02 ml/g, Rhone, Merieux, Harlow, UK) and then the animal was placed in a stereotactic frame with the head secured. A 1-cm midline incision was made to expose the skull after disinfecting the scalp. Thereafter, a 1-mm-diameter burr hole was drilled to implant a 0.5 ul syringe needle into the right striatum (stereotaxic coordinates: 0.2 mm anterior and 2.0 mm lateral to bregma, 3.5 mm in depth). Type IV bacterial collagenase (Sigma, St. Louis, MO, USA; 0.075 U in 0.4 ul of saline) was slowly injected into the striatum at a rate of 0.05 ul/min over 2 min with an infusion pump to induce intracerebral hemorrhage. Sham controls had only a needle insertion. The needle was left in place for an additional 10 min to avoid reflux before being slowly withdrawn. The incision was sutured after sealing the burr hole with bone wax. Body temperature was maintained through surgery until all mice recovered from anesthesia.

**Brain water content measurement**

The brain water content was determined by the wet-dry method^2^. Briefly, at 3 days after ICH, brain samples were harvested and divided into three parts (ipsilateral hemisphere, contralateral hemisphere, and cerebellum) and then weighed to obtain the wet weight. The samples were further placed into an 80 ℃ oven to dry for 5 days and reweighed to obtain the dry weight. The proportion of brain water content (%) was determined as followed: [(wet weight - dry weight)/wet weight] × 100%.

**Determination of the hematoma volume**

Hematoma volume measurement was conducted at 3 days after ICH onset. All mice were intracardially perfused with 40 ml ice-cold phosphate-buffered saline (PBS) under deep anesthesia. Afterward, the whole brains were carefully removed and sectioned into 1-mm-thickness coronal slices which were digitalized. The hemorrhage areas on each section were manually drawn and integrated along the anteroposterior axis to calculate the cubic volume of hematoma as followed: hematoma volume (mm^3^) = area of every section (mm^2^) × section number ×thickness (mm) by the ImageJ software.

**mNSS test**

The modified Neurological Severity Score (mNSS) test was performed blindly on day 1, day 3, day 7, and day 14 after ICH to examine neurological function^3^. The mNSS score ranges from 0 to 18, with a score of 0 indicating normal and higher scores implying more critical neurological impairment. The neurological function is mainly reflected in sensory (visual, tactile, and proprioceptive), motor (muscle status and abnormal movement), reflex, and balance tests.

**Corner turn test**

The corner turn test was conducted by two researchers who were blind to the whole experimental design to evaluate sensorimotor dysfunction after ICH operation^2^. Briefly, the mice were placed into a corner with a 30° angle assembled by two walls made of plastic fiberboards. The animal would generally turn left or right to escape the corner, and the options of direction were recorded after 10 repeated trials with 30 s intervals for each mouse. Only turns involving a full rearing along either wall were recorded. Generally, a mouse could turn either to the left or the right to exit the corner without turning bias, whereas a mouse suffering from ICH preferentially turns towards the unimpaired (i.e., right) body side. The percentage of right turns was calculated as followed: (the times of right turns/total trials’ times) × 100%.

**Measurement of intestinal barrier permeability**

The permeability of the intestinal barrier was investigated by assessing the circulating level of fluorescein^4^. Briefly, mice were fasted overnight before oral administration with 4 kDa fluorescein isothiocyanate (FITC)-dextran (FD4, 80 mg/ml, Cat#: FD4, Sigma-Aldrich). Blood samples were obtained 4 hours after FD4 gavage and centrifuged for 15 minutes (4000 rpm, 4 ℃). The upper supernatant was transferred into a new tube and used for serum FD4 concentration detection. Serum samples were used for FD4 assessment with a plate reader (excitation/emission 480 nm/530 nm). The circulating level of FD4 was detected in duplicate with the plate reader (excitation/emission 480 nm/530 nm) and calculated by establishing a standard curve.

**Immunofluorescence staining**

At 3 days and 14 days after ICH onset, brain and spinal cord samples were carefully isolated for histological analysis. Briefly, under deep anesthesia, mice were intracardially perfused with PBS followed by 4% paraformaldehyde (PFA). Brain and spinal cord samples were post-fixed in 4% PFA at 4 ℃ overnight and cryoprotected with 30% sucrose solution before snap-frozen using dry ice. Samples were sectioned into 10 um slices with a Leica CM1950 cryostat (Leica Biosystems Nussloch GmbH, Germany). Cryosections were incubated in blocking buffer containing 1× PBS, 5% normal goat serum (Cat#: 16210064, Thermofisher Scientific) and 0.3% Triton™ X-100 (Cat#: T8787, Sigma-Aldrich) at room temperature for 1 hour, and then incubated with primary antibodies [anti-MBP (1:400, Cat#: NB600-717SS, Novus), anti-NF200 (1:400, Cat#: N4142, Sigma-Aldrich), anti-GFAP (1:400, Cat#: E4L7M, CST), anti-CSPG (1:400, Cat#: C8035, Sigma-Aldrich), anti-Iba1 (1:300, Cat#: ab178846, Abcam)] at 4 ℃ overnight. Sections were rinsed three times in 1×PBS solution before incubated with fluorochrome-conjugated secondary antibodies [Alexa Fluor® 488 goat anti-mouse/rabbit antibody (1:400, Cat#: ab150113/ab150077, Abcam), Alexa Fluor® 555 goat anti-rabbit antibody (1:400, Cat#: ab150078, Abcam)] at room temperature for 1 hour. After being rinsed thrice, sections were counterstained with DAPI-containing mounting medium to visualize cell nuclei and imaged using a Nikon inverted microscope (ECLIPSE Ti2, Nikon Corporation, Japan).

**Fecal DNA extraction and 16S rRNA gene sequencing**

Fecal samples were collected on days 1, 3, and 14 after ICH. Bacterial DNA was extracted using the QIAamp Fast DNA Stool Mini Kit (Code No. 51604, Qiagen) according to the manufacturer’s instructions. Real-time quantitative polymerase chain reaction (RT-qPCR) was conducted for amplification of the 16S rRNA gene V4 hypervariable region with barcoded primers, forward primer (5′-GTGYCAGCMGCCGCGGTAA-3′) and reverse primer (5′-GGACTACNVGGGTWTCTAAT-3′). The PCR amplicons were mixed and sequenced with an Illumina MiSeq platform. The preprocessing of the raw sequences was performed using the BIPES protocol. A Quantitative Insights into Microbial Ecology 2 (QIIME2) workflow script was used to control the sequence qualification. Based on the determined sequence frequency, the operational taxonomic unit (OTU) clustering was performed with Usearch algorithm. BIOM files were generated using a QIIME2 workflow script, pick_closed_reference_otus.py. Alpha diversity was represented by the Shannon index, which indicated the abundance and distribution evenness of bacterial species. Beta diversity was estimated using the weighted UniFrac distances method. The smaller weighted UniFrac distances meant the higher similarity. Principal coordinates analysis (PCoA) was a dimensionality reduction method performed to visualize the relationship between samples based on the distance matrix. Linear discriminant analysis effect size (LEfSe) was an algorithm for high-dimensional biomarker discovery which was used to determine the discriminative characteristics between different groups. The linear discriminant analysis (LDA) score was applied to analyze the effect size of each differential abundance features. Microbial taxa with LDA score ≥ 3 were listed on the taxonomy bar plots and cladogram.

**Total RNA extraction and relative expression levels determine**

Total RNA of the brain and colon tissue was extracted using Trizol reagent (Code No. 9108, TaKaRa RNAiso Plus) following the manufacturer’s guidelines. The reverse transcription reaction was performed by using PrimeScript^TM^ RT reagent kit (Code No. RR037Q, TaKaRa) to generate cDNA, which was applied to subsequent quantitative PCR reaction using SYBR^R^ Premix Ex Taq^TM^ Ⅱ (Code No. RR820L, TaKaRa) on an Applied Biosystems ViiA^TM^ 7 Real-Time PCR system (Thermo Fisher Scientific, USA). The relative mRNA levels of target genes were calculated by the 2^-ΔΔCt^ method and normalized to the expression level of β-actin.

**Gut microbiota depletion and fecal microbiota transplantation**

Fecal samples were collected from the ICH+Vehicle group and ICH+OMT group at 3 days after ICH, then homogenized with sterile PBS (200 ul per 0.2 g feces) and centrifuged to obtain fecal suspension. To deplete gut microbiota, antibiotics mix (ampicillin 20 mg/ml, metronidazole 20 mg/ml, neomycin 20 mg/ml, and vancomycin 10 mg/ml, Sigma-Aldrich) were dissolved in sterile water and administrated by oral gavage for 1 week. The fecal microbiota transplantation (FMT) was conducted by daily oral gavage with 200 ul fecal suspension for 3 days after ICH surgery ^5^.

**Serum inflammatory cytokines and lipopolysaccharide measurement**

Venous blood samples were centrifuged for 15 minutes at 4 ℃ after natural coagulation. serum samples were transferred into a new tube for subsequent analysis. The serum levels of mouse interleukin-1β (IL-1β, Cat#: MM-0040M1), interleukin-6 (IL-6, Cat#: 0163M1), tumor necrosis factor-alpha (TNF-ɑ, Cat#: MM-0825M1) and lipopolysaccharide (LPS, Cat#: MM-0634M1) were measured using commercial ELISA kits (Meimian, China), according to manufacturer’s protocol.

1. Ng ACK, Yao M, Cheng SY, et al. Protracted Morphological Changes in the Corticospinal Tract Within the Cervical Spinal Cord After Intracerebral Hemorrhage in the Right Striatum of Mice. *Front Neurosci.* 2020;14:506.

2. Hua Y, Schallert T, Keep RF, Wu J, Hoff JT, Xi G. Behavioral tests after intracerebral hemorrhage in the rat. *Stroke.* 2002;33(10):2478-2484.

3. Zhao H, Pan P, Yang Y, et al. Endogenous hydrogen sulphide attenuates NLRP3 inflammasome-mediated neuroinflammation by suppressing the P2X7 receptor after intracerebral haemorrhage in rats. *J Neuroinflammation.* 2017;14(1):163.

4. Yu X, Zhou G, Shao B, et al. Gut Microbiota Dysbiosis Induced by Intracerebral Hemorrhage Aggravates Neuroinflammation in Mice. *Front Microbiol.* 2021;12:647304.

5. Gong S, Yan Z, Liu Z, et al. Intestinal Microbiota Mediates the Susceptibility to Polymicrobial Sepsis-Induced Liver Injury by Granisetron Generation in Mice. *Hepatology.* 2019;69(4):1751-1767.
